# Adult Stem Cell Transplantation: Is Gender a Factor in Stemness?

**DOI:** 10.3390/ijms150915225

**Published:** 2014-08-28

**Authors:** Naoki Tajiri, Kelsey Duncan, Mia C. Borlongan, Mibel Pabon, Sandra Acosta, Ike de la Pena, Diana Hernadez-Ontiveros, Diego Lozano, Daniela Aguirre, Stephanny Reyes, Paul R. Sanberg, David J. Eve, Cesar V. Borlongan, Yuji Kaneko

**Affiliations:** Center of Excellence for Aging & Brain Repair, Department of Neurosurgery and Brain Repair, University of South Florida College of Medicine, 12901 Bruce B. Downs Blvd., Tampa, FL 33612, USA; E-Mails: kduncan3@health.usf.edu (K.D.); borlongan.mia@tampaprep.org (M.C.B.); mpabon@health.usf.edu (M.P.); sacosta@health.usf.edu (S.A.); iked@health.usf.edu (I.P.); dhernan1@health.usf.edu (D.H.-O.); dlozano@mail.usf.edu (D.L.); daguirr1@health.usf.edu (D.A.); sreyes2@mail.usf.edu (S.R.); psanberg@.usf.edu (P.R.S.); deve@health.usf.edu (D.J.E.); cborlong@health.usf.edu (C.V.B.)

**Keywords:** menstrual blood, Sertoli cells, autologous, ischemic stroke, regenerative

## Abstract

Cell therapy now constitutes an important area of regenerative medicine. The aging of the population has mandated the discovery and development of new and innovative therapeutic modalities to combat devastating disorders such as stroke. Menstrual blood and Sertoli cells represent two sources of viable transplantable cells that are gender-specific, both of which appear to have potential as donor cells for transplantation in stroke. During the subacute phase of stroke, the use of autologous cells offers effective and practical clinical application and is suggestive of the many benefits of using the aforementioned gender-specific cells. For example, in addition to being exceptionally immunosuppressive, testis-derived Sertoli cells secrete many growth and trophic factors and have been shown to aid in the functional recovery of animals transplanted with fetal dopaminergic cells. Correspondingly, menstrual blood cells are easily obtainable and exhibit angiogenic characteristics, proliferative capability, and pluripotency. Of further interest is the ability of menstrual blood cells, following transplantation in stroke models, to migrate to the infarct site, secrete neurotrophic factors, regulate the inflammatory response, and be steered towards neural differentiation. From cell isolation to transplantation, we emphasize in this review paper the practicality and relevance of the experimental and clinical use of gender-specific stem cells, such as Sertoli cells and menstrual blood cells, in the treatment of stroke.

## 1. Introduction

Increased accessibility of stem cells has allowed for the advancement of a new arena in which to investigate many features of cell development including growth, proliferation, and differentiation. The fetus, embryo, and teratocarcinoma cells, as well as adult tissues, such as bone marrow, umbilical cord, placenta, and menstrual blood, have all been used as tissue sources of stem cells. Skin fibroblasts have also served as a source of stem cells as they have demonstrated the ability to exhibit pertinent characteristics such as prominent proliferative and differentiation potential in addition to neurotrophic and immunomodulatory secretory capabilities [1]. The use of stem cells for potential treatment of neurodegenerative, inflammatory, traumatic, and autoimmune disease processes has been investigated in the laboratory and tested in limited clinical trials. The preferred feature for stem cells utilized in transplantation therapy is their ability to avoid eliciting a response from the host’s immune system. As a result, the concept of autologous transplantation has become very attractive to many researchers in the field, instigating the exploration of gender-specific donor cells such as menstrual blood-derived stem cells and testis-derived Sertoli cells. For the purposes of this paper, “gender-specific cells” refers exclusively to Sertoli cells in males and menstrual blood-derived stem cells in females unless indicated otherwise. Menstrual blood and Sertoli cells have both been determined to be transplantable and, when used for the treatment of neurological disorders, have the benefit of being delivered either directly into the brain or peripherally by means of intravenous or intra-arterial administration.

In the United States of America, neurovascular diseases constitute the leading cause of chronic disability and the third highest cause of death [2,3,4,5]. In developed nations specifically, the radical transformation in lifestyle norms in recent generations and an aging population have contributed to the alarming prevalence of such diseases, including stroke [6]. Unfortunately, treatment for stroke has traditionally been very restricted as the only therapeutic option proven to result in improved clinical outcomes is tissue plasminogen activator (tPA), which has notable limitations in its application due to its exceptionally short therapeutic administration window of 4.5 h after symptom onset [7,8]. A report from 2012 estimates that utilization of tPA is as low as 4.1% of stroke patients in the United States [9]. These astounding figures necessitate the exploration and development of additional treatment alternatives. One such opportunity for advancement lies in reducing or eliminating secondary cell death [10,11,12,13], which is a common sequela of inflammation [14,15,16,17]. Both adult and embryonic stem cells appear to provide some degree of protective advantage from secondary cell death resulting from stroke [18,19,20,21].

Cells from menstrual blood retain the safety benefits inherent to the use of adult stem cells and display more immature phenotypes and behavior patterns than those observed in bone marrow-derived cells, thereby lending themselves towards fewer adverse effects, and with better outcomes [2,6,22,23]. As evidenced in laboratory studies using animal models of stroke, the ability of intravenously transplanted menstrual blood cells to migrate to the damaged brain tissue, release neurotrophic factors, and reduce inflammation has resulted in effective restoration of tissue and function in the central nervous system, heart, and ischemic limbs [23,24,25,26].

Similarly, the male-derived Sertoli cells have been demonstrated to enhance recovery from neurological disorders in animal models, such as Parkinson’s disease [27,28]. The impressive immunosuppressive attributes and release of trophic factors by Sertoli cells make them an excellent candidate for transplant, as these characteristics work to drastically reduce graft rejection and augment brain remodeling.

This review paper will distinguish both menstrual blood and Sertoli cells, present their respective strengths and modalities of brain repair and protection from injury secondary to stroke, and examine the potential use and practicality of both cell types within the realm of personalized medicine.

## 2. Disparities in Disease Prevalence in Men and Women

The fact that men are more prone to developing certain illnesses than women and *vice versa* with other illnesses is widely accepted, and stroke risk is no different. In 1974, an animal model validated the idea that susceptibility to stroke does indeed differ based on sex [29]. The study revealed that within a population of male and female rats with spontaneous hypertension, the portion of male rats that developed stroke was strikingly larger than the associated group of female rats who developed stroke. Later studies established a comparable pattern within human epidemiology for stroke [30] and cardiovascular disease [31].

Up to 75 years old, females have higher mortality rates from myocardial infarction and lower prevalence of stroke compared to males. This discrepancy between the genders regarding cardiovascular disease may be due in part to the cardiovascular protection afforded to females by oestrogens [32]. The preventative effects of aspirin also seem to be gender-dependent [33,34,35]. As evidenced by two gender-specific studies, aspirin has been proven effective in significantly limiting the occurrence of an initial myocardial infarction in men but not women. Comparable to this is aspirin’s ability to reduce the likelihood of a first stroke in females, but not in males. The aforementioned effects remain present even in prepubescent and postmenopausal populations where there is greater homogeny of hormones between males and females. This indicates that despite the fact that gonadal hormones probably do have an impact on differing incidences of certain disease processes among men and women, there is more to the equation.

Sex hormones such as testosterone and estrogen have been shown to influence cell survival and neurovascular protection as well. Oestrogen specifically has shown an ability to increase mitochondrial efficiency, suppress inflammation, and enhance vasodilator capacity, particularly with regards to cerebral vasculature [36]. The ability to limit inflammation following stroke coupled with its ability to impact vascular tone make oestrogen a hormone of interest for further study regarding stroke prevention and possible treatment.

## 3. Variations among Male and Female Cells

There are several general variations between male and female cells which must be acknowledged prior to proceeding to the more specific distinctions between menstrual blood and Sertoli cells. One of these is, of course, the existence of a Y chromosome in males which, despite containing repeats from X chromosomes, also codes for 27 proteins not found in females (and, therefore, obviously not present on X chromosomes), eight of which are expressed in the brain [37]. The human Y chromosome is the sex determining chromosome, with 196 proteins associated with this chromosome. Many of the genes and proteins present on the Y chromosome, likely also present in the brain, are thought to possess oncogenic and tumor suppressive effects, based on the observations that Y chromosomal mutations result in oncogenic disorders [37]. Many genes, and associated proteins, are unique to the Y chromosome, but genes in areas known as pseudoautosomal regions are present on both sex chromosomes, which function for normal development [37]. Of equal importance is the lack of male-specific minor histocompatibility antigens (such as Ubiquitously Transcribed Tetratricopeptide Repeat Containing, Y-Linked (UTY)) in female-derived cells [38], which has the potential to significantly influence the use of endometrial cells in stem cell-based therapies. Additionally, male X chromosomes do not have paternal imprinting, which, coupled with the previously mentioned dissimilarities, suggests the presence of noteworthy variations between male and female cells.

Recently published reviews indicate discrepancies in the ways male and female cells react under various conditions [39,40]. Results imply that female cells tend to be the more resistant of the two; however this can of course vary based on cause of injury. In reaction to ischemic injury, caspase-dependent cell death by way of activation of caspase 9 and 3 seems to be the tendency of female cells while male cells utilize a caspase-independent pathway. The method used by male cells involves making peroxynitrite ions and stimulating apoptosis inducing factor (AIF) and poly (ADP-ribose) polymerase (PARP) releasing peroxynitrite ions. Despite their apparently defensive role in male cells, PARP and nitric oxide synthase (NOS) inhibitors appear to be disadvantageous in females [41]. Interestingly, it appears that the reaction to ischemic insult within females does not include PARP and AIF at all, even though both seem to be just as active in females as in males [42]. On the other hand, the ischemic response to neonatal stroke in female rats was effectively diminished by caspase inhibitors, which was an effect not observed in their male counterparts [43]. The overexpression of P450 aromatase by astrocytes could provide a potential rationale to explain the disproportionate neuroprotection observed in females. This overexpression then permits the making of 17-estradiol, (a steroid with significant neuroprotective potential) which has been shown utilizing P450 aromatase inhibitor and conditioned media [44]. Interestingly, female animals display lower cell death from brain injury, such as stroke compared to age-matched males, likely due to neuroprotection by endogenous estradiol [43]. Similarly, female cells harvested from neonatal female brain exhibit better survival than male cells when exposed to experimental *in vitro* stroke models, in part due to enhanced aromatization and estradiol formation in female cells [44].

An equally important gender-specific cell phenotypic feature is the cell’s response to immune dysfunction, which is an established consequence of stroke in rat models [45,46,47]. Of note, most studies on rat stroke models documenting cell vulnerability to immunological insults used male rats, but similar results were found in female rats following removal of their ovaries [48]. An in-depth examination of the cellular component responsible for such gender-specific vulnerability to aberrant alterations in the immune system, reveals the key participation of the female cells to harbor estradiol and/or estrogen receptors, in that treatment with estradiol and/or estrogen receptor agonist was shown to repair the immune systems of the ovariectomized rats, purportedly due to the neuroprotective capabilities of estradiol in stroke by means of interleukin-1β expression [49] and neurogenesis [50]. However, the female cells’ response to such hormonal therapy is influenced by aging, as some evidence exists to suggest that estrogen can have a harmful impact on postmenopausal females in animal models, possibly due to the presence of less insulin-like growth factor (IGF) in older animals [51]. Accordingly, a careful examination of the cell’s gender and its associated receptor and growth factor component is vital to understanding the cell’s response to immune dysfunction and brain insults.

## 4. Dissimilarities between Male- and Female-Derived Stem Cells

Stem cell activity can vary significantly by gender. For example, mesenchymal stem cells (MSCs) from two year old female Rhesus monkeys were evidenced to have greater neurogenic capacity than MSCs from male monkeys [52]. In regards to neural fate and steroid receptors, neural stem cells (NSCs) obtained from young and old rats exhibited sexual dimorphism. Male cells preferentially differentiate into a neuronal or oligodendroglial fate, while cells from females have a propensity for an astrocytic lineage [53].

Haematopoietic stem cells in mice have been evidenced to exhibit differences in cell-cycle regulation according to gender as a result of oestrogen. This causes the haematopoietic stem cells of female mice to divide much more frequently than those of their male counterparts. During pregnancy, oestrogen levels increase, resulting in increased frequency of haematopoietic stem cell division. Haematopoietic stem cells also divided more frequently following administration of oestradiol, a hormone primarily found in the ovaries. The testis also produces oestradiol and this suggests some hormonal similarity between male and female cells. This effect was true for both male and female mice that received oestradiol [54].

To further evidence the variations between male and female stem cells, one retrospective study looking at more than 50,000 patients who had received allogenic haematopoetic stem cell transplants showed that male patients who had been matched with female donors had worse outcomes than any other patient/donor gender combination. Specifically, female donor/male recipient patients experienced an increased rate of graft-*versus*-host disease (GvHD) and transplant related mortality (TRM). These patients also appeared to have a reduced risk of relapse, however this was not enough to offset the mortality associated with higher incidence of GvHD and TRM [55].

Additionally, the cytokine expression of MSCs harvested from the bone marrow of male mice has been observed to have higher concentrations of interleukin-6 (IL-6) and tumor necrosis factor (TNF) than cells derived from female cells. The opposite effect was noted with anti-inflammatory vascular endothelial growth factor (VEGF), which is higher in female cells than male cells [56]. The male cells also appear to have an increased predisposition for hypoxia *in vitro*. Better functional recovery, decreased levels of TNF, and more VEGF were observed subsequent to transplantation of MSCs from female mice into isolated rat hearts after ischemia when evaluated against male MSC transplantation [57]. Following the transplantation of mononuclear cells (MNCs) from female bone marrow into male atherosclerotic apolipoprotein E (ApoE) mice, there was a reallocation of cytokines from proinflammatory to anti-inflammatory and haematopoeitic regulatory cytokines. This event corresponded with a decrease in plaques associated with atherosclerosis, whereas cells derived from male bone marrow did not demonstrate the same beneficial capacity. A greater improvement in endogenous repair was detected following the same procedure in female atherosclerotic ApoE mice than that observed in male mice [58], however, no additional atheroprotection was discerned [59].

Another gender-based difference is the overexpression of estrogen receptor alpha (ERα) in young male-derived cells, whereas estrogen receptor beta (ERβ) is the more prevalent receptor in young female cells [53,60]. Uncertainty remains regarding the presence of ERβ on Sertoli cells. ERα appears to be favored in humans [61] however reportedly trace expression of ERβ implies the reverse [62]. ERβ has emerged as being more prevalent than ERα in baboons, which were also discovered to express aromatase [62,63]. Whether or not menstrual blood contains either of these receptors is yet to be determined, however, it has been established that ERβ is the prevailing receptor for endothelial cells of the endometrium, whereas both ERα and ERβ are expressed by the perivascular cells [64]. The differences in receptor types between the male and female cells is of particular interest due to the impact of ER expression in the brain, as ERα is thought to have a significant influence on neuroprotection and ischemic risk [65].

In one study, following transplantation of NSCs from young and old rats of both genders into rats of both sexes and age categories (young and old), young rats were noted to have a poorer cell survival rate while also displaying improved neurogenesis. Older animals had better cell survival both with cells derived from younger, same-sex rats and cells derived from older, opposite-sex rats [66]. This indicates the potential for benefit utilizing both autologous and allogenic transplantation while accentuating the significance of the age and gender of both donor and recipient. Despite some recent analysis on the possible consequences of sexual dimorphism on NSCs [67], the possible therapeutic impact of gender-specific cells still lacks adequate investigation.

It is suggested that anti-inflammatory cytokines might be generated by stem cells found in menstrual blood, a theory which is supported by the fact that when these stem cells were introduced into mixed lymphocyte cultures, IL-4 was activated while TNF was repressed following endotoxin stimulation [25]. Additional supporting evidence comes from their important contribution to tissue replenishment and angiogenesis necessary for endometrial sloughing during menstruation. Sertoli cells have evidenced their own inherent benefits including supporting tolerance following cotransplantation [68] and the release of proangiogenic factors [69]. Based on these studies, it is probable that both stem cells from menstrual blood and Sertoli cells could play a valuable role in immunomodulation and angiogenesis. The stemness of Sertoli cells and the current areas within this field of study require further investigation.

## 5. Characterization of Gender-Specific Donor Cells for Transplantation

### 5.1. Transplantable Qualities of Endometrial Cells

The existence of stem cells in the endometrium was initially explained over 35 years ago [70]. As a result of the concept of regular sloughing of the endometrium, cells with prominent capabilities for proliferation were identified [71]. In opposition to the original idea that stem cells could only be obtained from the basalis layer of the endometrium, stem cells were found [71] and confirmed [22,72,73] to be present in menstrual blood. Clonogenic and proliferative capabilities were both observed when stromal and epithelial cells were cultured *in vitro* (following isolation from menstrual blood or the endometrium). Although it must be noted that the phenotypic markers found on the epithelial cells quickly begin to diminish and the cells require a nutrient layer in order to stay alive [71,74]. In addition to clonogenicity, stromal stem cells derived from menstrual blood (MenSCs) displayed multipotentiality and the ability to grow *in vitro* [22]. Octamer binding transcription factor 4 (Oct-4), stage specific embryonic antigen 4 (SSEA-4), and v-kit Hardy-Zuckerman 4 feline sarcoma viral oncogene homolog (c-kit), all of which are markers of pluripotency typically observed in immature cell varieties, such as embryonic stem cells, are seen on MenSCs. Recent investigation of cells from human menstrual blood has suggested that these cells not only have a high rate of proliferation, but also exhibit embryonic cell indicators, contributing to their immature phenotype [72]. In addition, the cells stayed unchanged even following 20 culture passages and have the ability to undergo subculturing up to 47 times prior to senescence, indicating great potential for viability and longevity.

A recent study found that menstrual blood-derived MSCs show potential as a cell source for nuclear reprogramming. Following transduction with recombinant retroviruses expressing the coding regions of the genes OCT4, sex determining region Y box 2 (SOX2), and Kruppel-like factor 4 (KLF4), the MSCs efficiently and effectively produced induced pluripotent stem cells (iPSCs). This method proved to be both cost-effective and faster (only taking about 15–17 days) than the traditional method of forced ectopic expression of v-myc avian myelocytomatosis viral oncogene homolog (c-MYC), OCT4, SOX2, and KLF4 in fibroblasts [75].

In addition to convenience in clinical application, a study of transplantation of menstrual blood derived stem cells into an *in vivo* adult rat stroke model suggests that the use of these cells can have a beneficial impact on behavioral and histological deficits following stroke [24]. Moreover, the accompanying *in vitro* study seems to indicate that the cells offer the benefit of an embryonic-like stem cell phenotype and a notable degree of protection from ischemic insult while also having the ability to be steered into a neural lineage. The improvement in neurological deficits noted with the use of these cells could be of tremendous benefit to those who suffer from stroke, as even a fairly minimal improvement in cognitive and behavioral function can mean the difference between independent living and institutionalization for many individuals [76].

### 5.2. Transplantable Qualities of Sertoli Cells

Sertoli cells derived from the testis have a variety of roles, including spermatogenesis [77] and the formation of the blood-testis barrier [78]. They have displayed an ability to supplement the treatments for many disease processes including Parkinson’s Disease, Huntington’s Disease, and type 1 Diabetes, in addition to assisting with allogenic and xenogenic engraftment [69,79,80,81,82].

The release of trophic factors and contribution to tissue remodeling are both known functions of Sertoli cells. Sertoli cells also aid in the development of testis and germ cells via the circulation of nutritive factors and regulatory proteins [27,83,84]. The impact of Sertoli cells on the viability of germ cells is of utmost importance in preventing an immune response and potential rejection [69], making the testes an immunoprivileged area [27]. Sertoli cells are believed to be involved in the secretion of the cluster of differentiation 95 (CD95) ligand [85] and Fas ligand (FasL) [86,87], both of which participate in immunosuppression. Additionally, a recent *in vivo* islet transplantation study that investigated the impact of endothelial cell coating and Sertoli cell infusions on vascularization and graft rejection inhibition in rats has indicated that Sertoli cells also play a part in limiting lymphocyte activation and inflammatory cytokines [85]. Glial cell line-derived neurotrophic factor (GDNF), which is believed to assist in the regeneration of spermatogonial stem cells, is another factor released by Sertoli cells [88,89], while multiple sources also implicate Sertoli cells in the secretion of angiogenic factors [85,90] and the promotion of neovascularization [69].

It must also be noted that one study found that mouse fibroblasts could be reprogrammed into embryonic Sertoli-like cells following concomitant expression of the transcription factors nuclear receptor subfamily 5, group A, member 1 (Nr5a1), Wilms tumor 1 (Wt1), doublesex and male abnormal-3 (mab-3) related transcription factor 1 (Dmrt1), guanine-adenine-thymine-adenine binding protein 4 (Gata4), and sex determining region Y box 9 (Sox9). This is significant because it has the potential to promote continued research regarding the therapeutic applications and functions of Sertoli cells by providing a source of available cells with reliable and predictable characteristics for use in experimentation [91].

## 6. Experimental and Clinical Applications of Gender-Specific Stem Cells

### 6.1. Endometrial Cell Transplantation Studies

The use of myoblasts impregnated with endometrial and menstrual blood cells in a murine model of Duchenne muscular dystrophy were shown to assist in the generation of human dystrophin within the muscle [73]. Menstrual blood cells have also been found to provide neuroprotection in cultured rat neurons exposed to hypoxic damage [24]. These cells also led to enhanced neurological functioning when utilized in a rat stroke model independent of administration site (local *vs.* systemic) [24]. Tissue examination in the aforementioned studies found that human cells had moved to parts of the rat brain that had not been injected [24]. The lack of evidence of differentiation indicates that the primary method of restoration in brain tissue is not differentiation [24].

Using a Parkinson’s disease mouse model, stromal cells obtained from the endometrium differentiated into dopamine-like cells that expressed tyrosine hydroxylase (TH) [26], which is the precursor of dopamine. These cells migrated to the substantia nigra where they developed a neural phenotype and started producing TH in order to assist in the healing of injured tissue.

In one study that examined the safety of using stromal cells from the endometrium, four multiple sclerosis patients were injected with 16 to 30 million cells intrathecally [92]. One patient did receive a second dose of cells. No undesirable effects were noted, and all patients remained functionally stable a year after administration [92].

Cells derived from the endometrium exhibit a powerful aptitude for angiogenesis, which is probably a result of the cells’ inherent roles of proliferation and embryonic implantation within the endometrium [93]. It has been proposed that these cells, in conjunction with cells utilized in intelligent artificial films [94], could be developed as a therapeutic option for acute burns to the skin and chronic limb ischemia due to their strong angiogenic capabilities.

### 6.2. Sertoli Cell Transplantation Studies

Sertoli cells have demonstrated the ability to promote DA-secreting adrenal chromaffin cell survival, causing an almost imperceptible immune response within the central nervous system (CNS) [95,96]. They have also been found to exert a neuroprotective effect in 6-hydroxdopamine-induced hemiparkinsonism within a rat model [27,28]. Sertoli cells were introduced into both male and female rats in order to see whether hormones or gender play a part in their effectiveness [27]. The outcome revealed that the functional improvement of the male and female hemiparkinsonian rats were comparable to one another, refuting the ideas that the effects of Sertoli cells are gender-dependent [95,96] or influenced by testosterone.

Sertoli cells have been evidenced to assist with immunosuppression post-transplantation by utilizing the FasL pathway [86,87]. These effects are noted even without supplementary immunosuppressive measures [96,97] as both allografts and xenografts lived without any systemic immunosuppression for two months after transplantation [28]. Sertoli cells also secrete nutritive, trophic, and regulatory proteins, such as sulfated glycoprotein-2, androgen binding protein, epidermal growth factor, transforming growth factor-a and -b, insulin-like growth factor, fibroblast growth factor [84,98], and VEGF [99], all of which can aid in graft survival. Optimally, both Sertoli and menstrual cells would be harvested and banked prior to any damage, as the cells could then be thawed at a later date for autologous transplantation. Table 1 provides a *vis-à-vis* comparison between Sertoli cells and menstrual blood cells in terms of cell fate, trophic factors released, targeted diseases, functional outcomes, and general advantages and limitations to allow a better appreciation of the two gender specific cells.

**Table 1:** A *vis-à-vis* comparison of gender-specific cells.

Stem Cell Features	Sertoli Cell	Mentrual Blood Cells
Cell fate	Phenotype retained	Neural phenotype
Therapeutic molecules	Immunosuppressant factors	Trophic factors
Disease target	Parkinsonʼs disease	Stroke, limb ishemia
Functional outcome	Better co-graft survival	Reduced host cell loss
Cell therapy use	Localized immunosuppressioin or by-stander effects	Cell replacement or by-stander effects
Limitations	Pre-pubertial harvest	Menstruation period harvest

## 7. Practical Aspects of Personalized Medicine with Emphasis on Gender-Specific Stem Cells

**Figure 1:**
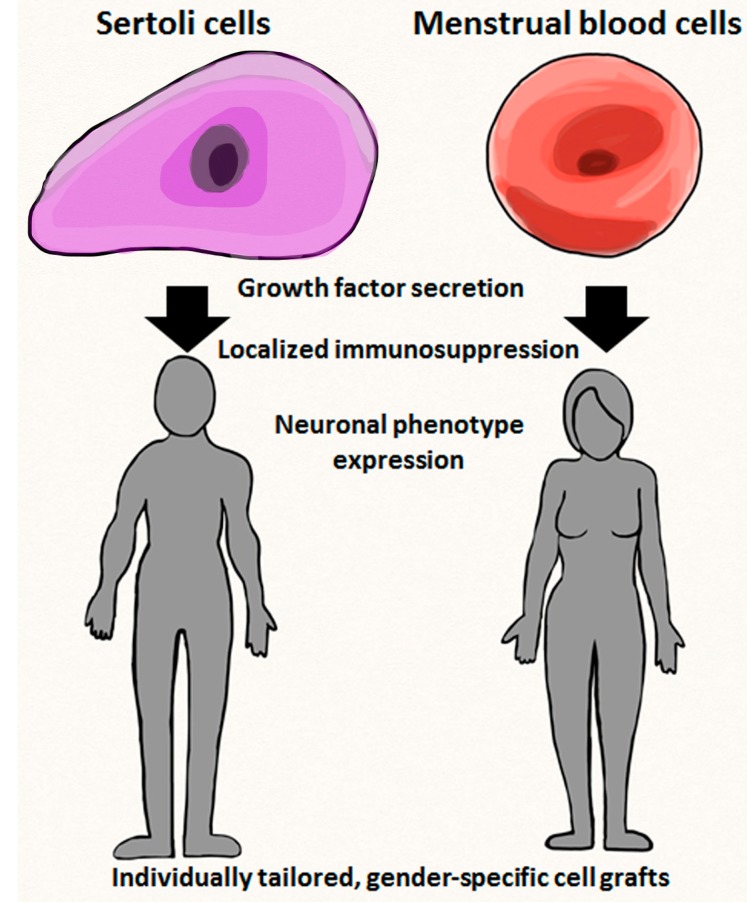
Sertoli cells and menstrual blood cells. Figure shows potential gender effects on stem cell biology such as growth factor secretion (both cells), localized immunosuppression (Sertoli cells) and neuronal phenotype expression (menstrual blood cells), as well as their therapeutic outcomes (*i.e.*, individually tailored, gender-specific cell grafts).

The stem cell arena has been evolving and expanding at an astonishing rate and may play a key role within personalized medicine (Figure 1). Stem cells and the field of cellular reprogramming have created many possibilities for researchers, including the correction of genetic mutations, regenerative medicine, and the potential to develop treatments and cures for a variety of disease processes including cancer [100].

Menstrual blood cells may offer significant potential within the realm of personalized medicine. Due to decreased ethical issues, safety concerns, and reduced possibility of an immune reaction/transplant rejection, autologous transplantation remains the ideal. While both menstrual blood cells and bone marrow-derived cells have the capacity for autologous transplantation, the utilization of menstrual blood cells is superior to that of bone marrow-derived cells for several reasons. For example, menstrual blood cells are much easier to collect and bank for future use than bone marrow-derived cells. They also have a superior capacity for expansion (up to 68 doublings without evidence of karyotypic or functional abnormalities) and can be pre-differentiated into patient-specific tissues which can be placed on hold until needed [71]. Umbilical cord blood banking has been available for quite some time, but menstrual blood banking is only in its infancy. Females of child-bearing age are permitted to donate many times which allows for the banking of a considerable quantity of cells. These cells are then available for expansion, differentiation, and transplantation when they are needed at a later date [101].

There is a high likelihood for Sertoli cells within personalized medicine as well, although the fact that they can only be obtained from pre-pubescent donors may become a somewhat limiting factor. The proliferative capacity of Sertoli cells decreases with age. Around puberty, the traditionally accepted concept is that immature, proliferating Sertoli cells transition into post-mitotic, terminally differentiated cells [102], thereby necessitating that cell harvest takes place prior to the onset of puberty. However, this terminal differentiation of adult Sertoli cells has been challenged in that they are able to dedifferentiate and retain proliferative ability in rodents and humans. Data over the last decade raises the question of stem cell progenitor cells in the Sertoli cell population, which provide the basis for the therapeutic use of Sertoli cells in clinicial settings [103].

Sertoli cells also demonstrate immunosuppressive properties and were capable of lowering blood glucose in diabetic mice [104]. In one study, spermatogonial stem cells harvested from young males suffering from leukemia and at risk for sterility were autologously transplanted back into patients following intensive oncological treatment, reducing their probability of sterility in adulthood [105]. Hopefully, a comparable treatment will eventually be available for stroke, whereby Sertoli cells will be harvested from patients at risk for stroke which can then be transplanted back into the patient directly following ischemic stroke.

## 8. Limitations Regarding Gender-Specific Donor Cells

Several limitations to stem cell therapy exist. A lack of stem cell donors, risk of infection, age restrictions, and methods of banking and storage are all potential hazards and limitations associated with stem cell use. Often, the idea of banking menstrual blood cells is not very appealing to women who find the harvesting process unpleasant and may not be able to see any immediate advantage to doing so. Some females also postpone having their cells collected because they expect to continue menstruating for several decades and see no reason to undergo the procedure sooner rather than later. An additional consideration is the fact that the transplantation of menstrual blood is confined to females only in many situations.

As of yet there is no type of male stem cell that can quite parallel the cells obtained from menstrual blood. There are drawbacks and restrictions associated with all types of stem cells, the most prevalent of which in Sertoli cells being the relatively small window of opportunity for harvesting and their inability to transdifferentiate [106,107,108,109,110,111,112]. ERα knockout and aromatase-deficient female mice over time display ova-testis with the transdifferentiation of granulosa cells to Sertoli cell-like cells [113]. Finally, despite some exploration of Sertoli cell transplants into female rats [27] and xenografted Sertoli cells [28], the use of Sertoli cells in males diagnosed with neurodegenerative disease, particularly via autologous transplantation, remains an area requiring further exploration.

## 9. Conclusions

Promoting survival and functional recovery following stroke is of utmost importance and appears to be an area in which cell-based therapy has a lot of potential [4]. By modulating the immune system and releasing trophic factors, stem cells have demonstrated significant promise in helping to restore the brain post-stroke [16]. The use of numerous therapeutic factors (such as those with anti-inflammatory and immunosuppressive capabilities) within cell-based stroke treatment looks promising. Differentiation of stem cells (albeit towards neural lineage) has indeed been evidenced by multiple studies; however the prerequisite of differentiation to the overall goal of brain repair is still a subject of debate. Importantly, harvest of autologous cells and their subsequent storage via cryopreservation may be important transplantable donor cell criteria for stoke. While logistical difficulties and questions relevant to their mechanism of action still exist, translational studies directed at optimizing the therapeutic outcomes of menstrual blood and Sertoli cells will further clarify the potential of gender-specific stem cells as effective donor cells for transplantation therapy in stroke.
